# Intraparenchymal infiltration of Rathke’s cleft cysts manifesting as severe neurological deficits and hypopituitarism: 2 case reports

**DOI:** 10.1186/s13104-016-2035-1

**Published:** 2016-04-19

**Authors:** Yoshikazu Ogawa, Mika Watanabe, Teiji Tominaga

**Affiliations:** Department of Neurosurgery, Kohnan Hospital, 4-20-1 Nagamachiminami, Taihaku-ku, Sendai, Miyagi 982-8523 Japan; Department of Pathology, Tohoku University Graduate School of Medicine, 1-1 Seiryo-machi, Aoba-ku, Sendai, Miyagi 980-8574 Japan; Department of Neurosurgery, Tohoku University Graduate School of Medicine, 1-1 Seiryo-machi, Aoba-ku, Sendai, Miyagi 980-8574 Japan

**Keywords:** Infiltration, Intraparenchymal, Rapid deterioration, Rathke’s cleft cyst, Pulse steroid therapy

## Abstract

**Background:**

Rathke’s cleft cysts generally remain asymptomatic throughout life, but a few patients may suffer severe neurological and/or endocrinological deficits. The symptoms include visual disturbances caused by compression of the optic chiasm, and severe endocrinological deficits caused by repeated intracystic hemorrhage or leakage of cyst content. However, no case of Rathke’s cleft cyst has infiltrated into neuroglial tissue with marked cerebral edema.

**Case presentation:**

Two patients presented with non-infectious re-deterioration of Rathke’s cleft cysts with intraparenchymal infiltration and marked cerebral edema, to ipsilateral hypothalamus in one case and to the bilateral frontal lobes in the other. Both patients were surgically treated by extended transsphenoidal surgery, and showed remarkable improvement with postoperative pulse-dose steroid therapy, including disappearance/shrinkage of abnormal enhanced lesion and cerebral edema on magnetic resonance imaging. Histological examination disclosed significant squamous metaplasia in epithelia and marked infiltration of inflammatory cells into the pituitary gland and neuroglial tissues. Most infiltrated cells were lymphocytes and plasma cells, thought to indicate the involvement of long-term underling inflammatory processes in this phenomenon.

**Conclusion:**

Long-term subclinical inflammation may be the mechanism of this extraordinary aggressive clinical course. Postoperative steroid administration should be reduced prudently, and careful follow-up imaging is essential in cases of Rathke’s cleft cyst with abnormal histological findings.

## Background

Rathke’s cleft cyst is considered to arise from the remnants of Rathke’s diverticulum or Rathke’s pouch, which is formed at 4–6 weeks of gestation, and consists of single cuboidal or columnar epithelium including cilia and goblet cells, which secrete mucus into the cyst. Most of these cysts remain asymptomatic throughout life, but a few patients may suffer severe neurological and/or endocrinological deficits and require surgical intervention [1, 2]. The symptoms include visual disturbances caused by expansion and compression of the optic chiasm, and severe endocrinological deficits caused by repeated intracystic hemorrhage or leakage of cyst content [1, 2]. Treatment generally consists of partial cystectomy and subsequent aspiration of the mucus at the initial operation [3, 4], although intracystic fixation of the cyst wall with pure ethanol is sometimes preferred [3, 5]. Around 90 % of surgically treated cases are reported to achieve remission [6, 7], but a few distinctive cases have developed repeated re-accumulation and became intractable [3, 8, 9]. However, no case of Rathke’s cleft cyst associated with intraparenchymal infiltration has been reported.

We describe 2 cases of re-deteriorated Rathke’s cleft cyst, which had infiltrated into the neuroglial tissue with marked cerebral edema, treated by aggressive removal and long-term steroid administration.

## Case presentation

### Case 1

**Fig. 1:**
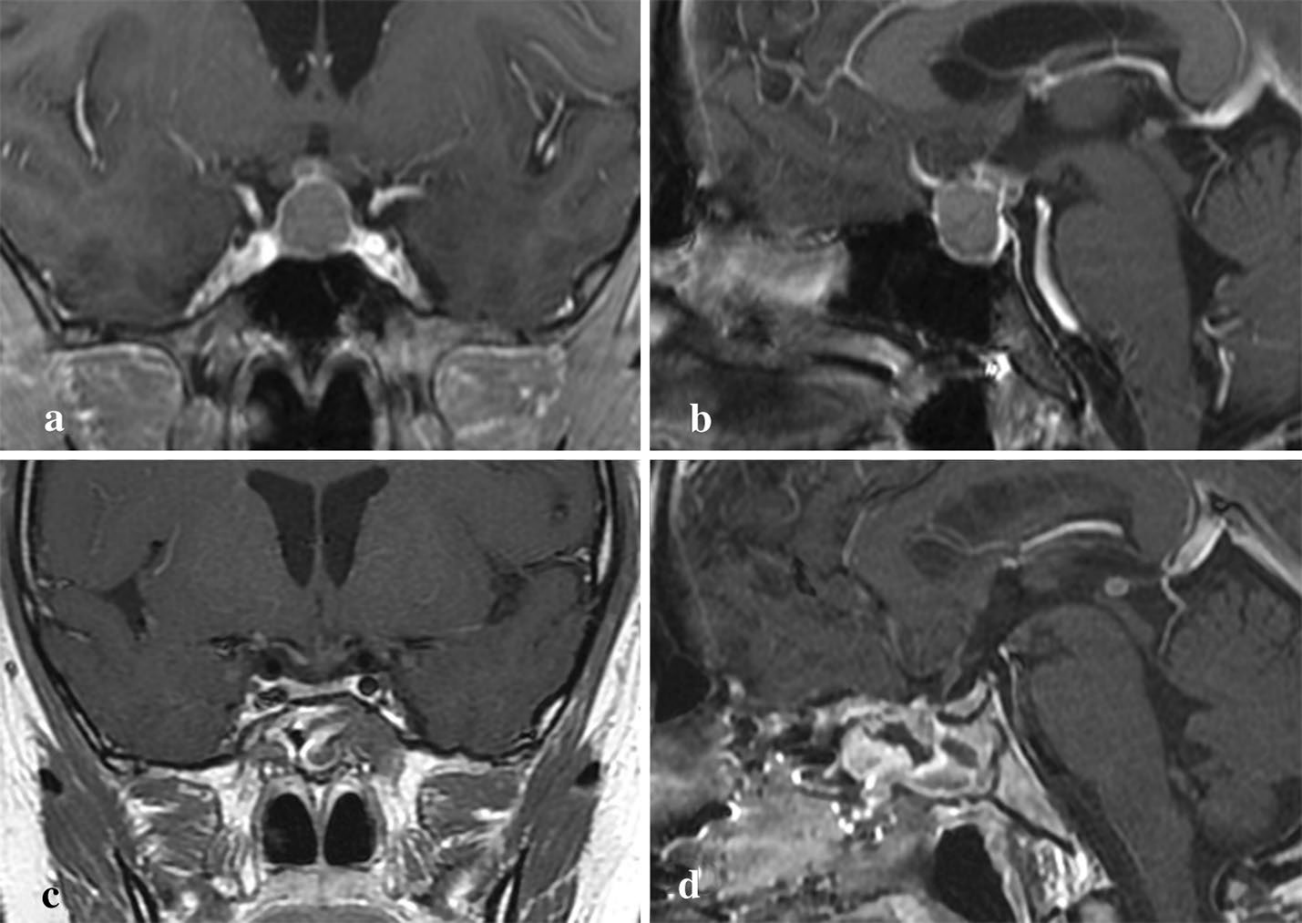
Case 1. **a** Preoperative coronal T1-weighted MR image with gadolinium revealing a large cystic lesion extending from the sella turcica to suprasellar cistern. **b** Preoperative sagittal T1-weighted MR image with gadolinium revealing than this cyst was multicystic. Cysts were collapsed after the operation (**c**, **d**)

A 36-year-old Japanese woman was referred to our hospital with re-deteriorated visual disturbance. She had a long history of schizophrenia, which was well controlled by a psychologist. She had suffered visual disturbance caused by Rathke’s cleft cyst 6 months previously, and severe hypopituitarism was discovered. Transsphenoidal surgery was performed with aspiration of cyst content and ethanol fixation of the wall (Fig. 1). Cyst cavity did not communicate with cerebrospinal fluid (CSF) spaces. She was discharged without neurological deficits but hypopituitarism persisted, which was supplemented with daily administration of dexamethasone 0.25 mg and levothyroxine 50 μg.

**Fig. 2:**
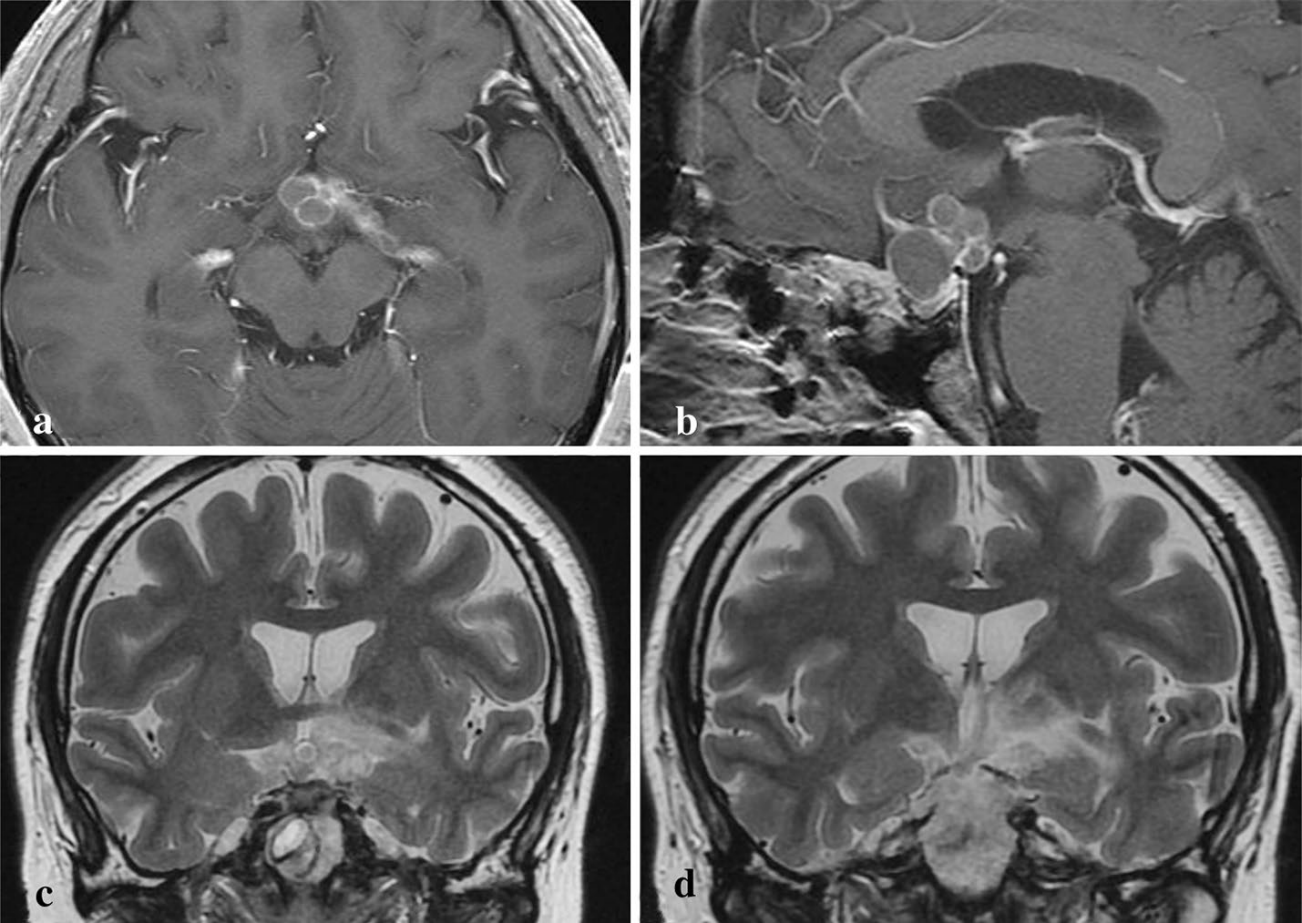
Case 1. **a** Preoperative axial T1-weighted MR image with gadolinium revealing a multicystic sellar lesion extending from the suprasellar cistern to the left ambient cistern. **b** Preoperative sagittal T1-weighted MR image with gadolinium revealing infiltration of the lesion to the third ventricle floor. (**c**, **d**) Preoperative coronal T2-weighted MR images revealing an extensive irregular high intensity area in the left hypothalamus

On admission her consciousness was clear, and body temperature was 36.4 °C. Peripheral white blood cell count was 5200/μl and C-reactive protein level was 0.08 mg/dl. All other clinical tests were negative for systemic inflammation or infection. Humphrey visual field analyzer examination showed temporal hemianopsia of the right eye and whole visual field defect of the left eye. Magnetic resonance (MR) imaging demonstrated a multicystic lesion compressing the optic chiasm upwards, and extending from the suprasellar cistern to the left ambient cistern. Part of the lesion had clearly infiltrated into the left hypothalamus, and extensive cerebral edema was present (Fig. 2).

**Fig. 3:**
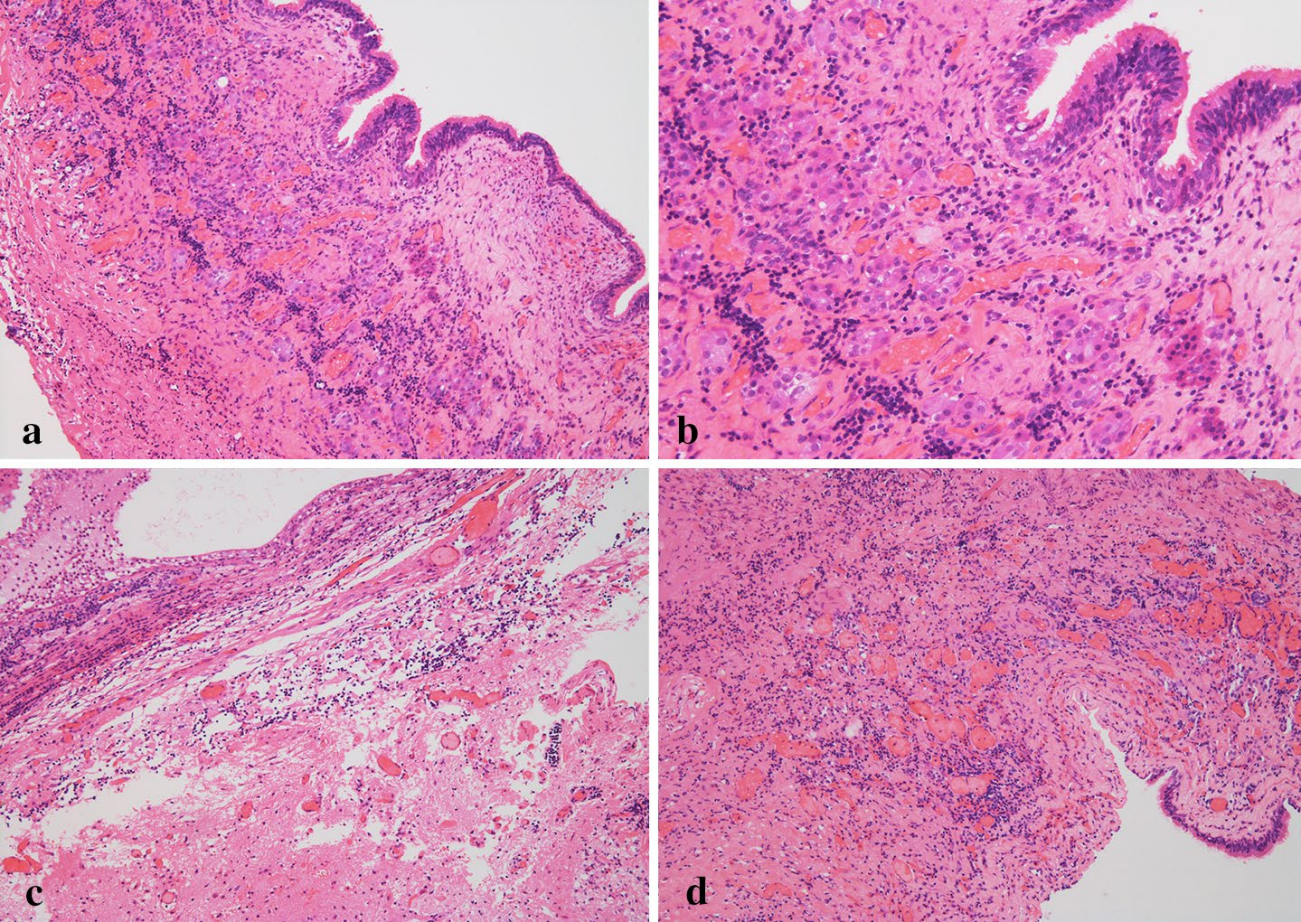
Case 1. **a** Photomicrograph showing smooth transition from single columnar epithelium to stratified squamous epithelia, original magnification ×100. **b** High-power photomicrograph showing involvement of the pituitary gland in sub-epithelial inflammatory granulation. Most infiltrated cells were lymphocytes and plasma cells, original magnification ×200. **c** Inflammatory cells had frequently infiltrated into the arachnoid and neuroglial tissues, original magnification ×100. **d** Significant angiogenesis is present, original magnification ×100. Hematoxylin and eosin staining

Surgery was performed through the extended transsphenoidal approach. Intraoperative finding disclosed the tough and thick rugged lesion, which was removed en-bloc together with the infiltrated third ventricle floor. Subtotal removal was achieved. Postoperative histological examination disclosed ciliated and goblet cell columnar epithelium, containing a small component of squamous metaplasia in the lining. The diagnosis was established as Rathke’s cleft cyst. However, significant sub-epithelial infiltration of inflammatory cells was seen, and massive inflammatory granulation had formed. Most infiltrated cells were lymphocytes and plasma cells, frequently seen in neuroglial tissues, and the pituitary gland was isolated in this inflammatory ocean (Fig. 3).

Rapid deterioration caused by immune-response abnormality was suspected and pulse-dose steroid therapy was begun with daily methylprednisolone 1000 mg for 3 days and gradual reduction to daily dexamethasone 0.5 mg with a short bridge of hydrocortisone. She was discharged after 22 postoperative days without abnormal finding on MR imaging or neurological deficit. She remained in good condition with supplements of daily triamcinolone 2 mg and levothyroxine 50 μg after 26 months.

### Case 2

**Fig. 4:**
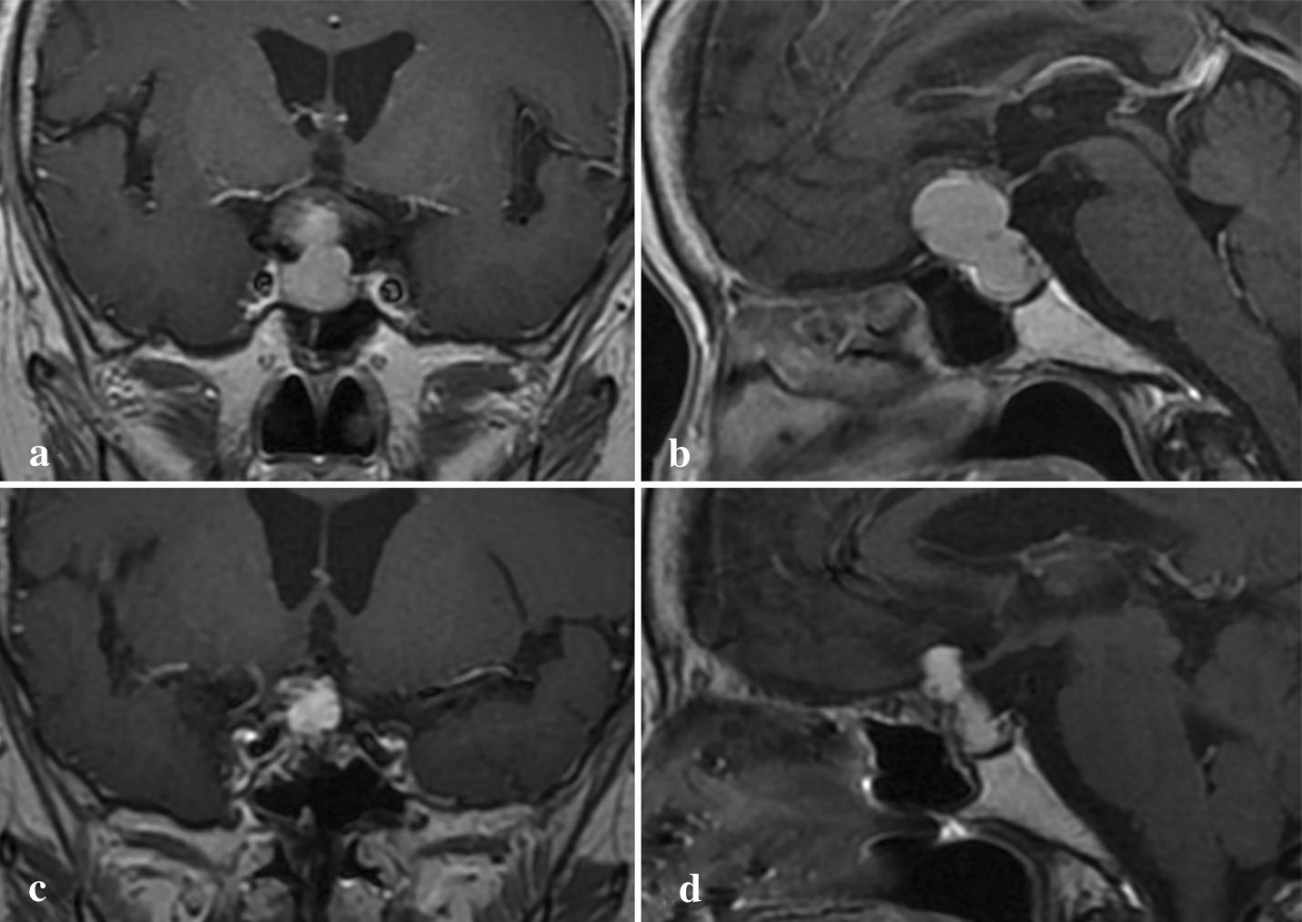
Case 2. **a** Preoperative coronal T1-weighted MR image with gadolinium revealing a dumbbell-shaped cystic lesion extending from the sella turcica to suprasellar and cistern. **b** Preoperative sagittal T1-weighted MR image with gadolinium revealing the cyst compressed optic chiasm upwards. Cyst was almost collapsed after the operation (**c**, **d**)

A 67-year-old Japanese woman was referred to our hospital due to disturbed consciousness with urinary incontinence and severe fatigability. She had suffered visual disturbance caused by Rathke’s cleft cyst, and severe hypopituitarism was identified 18 months previously. Transsphenoidal surgery was performed with aspiration of cyst content and ethanol fixation of the wall (Fig. 4). Cyst cavity did not communicate with CSF spaces. She was discharged without neurological deficits but hypopituitarism persisted, which was supplemented with daily dexamethasone 0.25 mg.

**Fig. 5:**
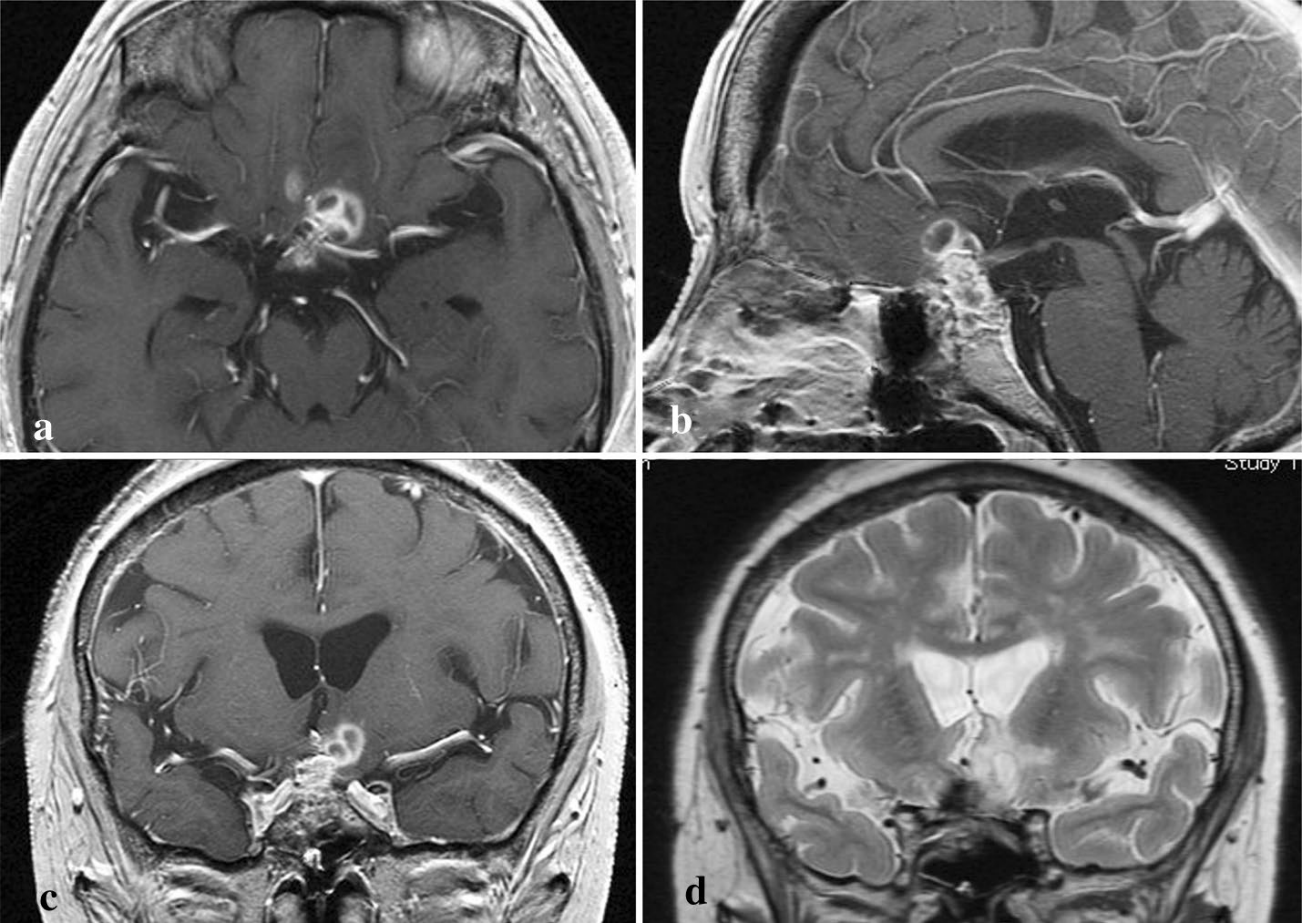
Case 2. Preoperative axial (**a**) and sagittal (**b**) T1-weighted MR images with gadolinium revealing a multicystic suprasellar lesion infiltrating into the bilateral frontal lobes. **c** Preoperative coronal T1-weighted MR image with gadolinium revealing infiltration of the lesion into the left rectal gyrus. **d** Preoperative coronal T2-weighted MR image revealing an extensive irregular high intensity area in the left frontal lobe

On admission her consciousness was 14 (E4, V4, M6) on the Glasgow Coma Scale, and body temperature was 36.0 °C. Peripheral white blood cell count was 9000/μl and C-reactive protein level was 0.63 mg/dl. All other clinical examinations were negative for systemic inflammation or infection. Humphrey visual field analyzer examination showed temporal hemianopsia of the right eye and upper temporal quadrantanopsia of the left eye. MR imaging showed a ring-shaped well-enhanced lesion in the suprasellar cistern, which had infiltrated into the bilateral frontal lobes. Extensive cerebral edema in the left frontal lobe was also present (Fig. 5).

**Fig. 6:**
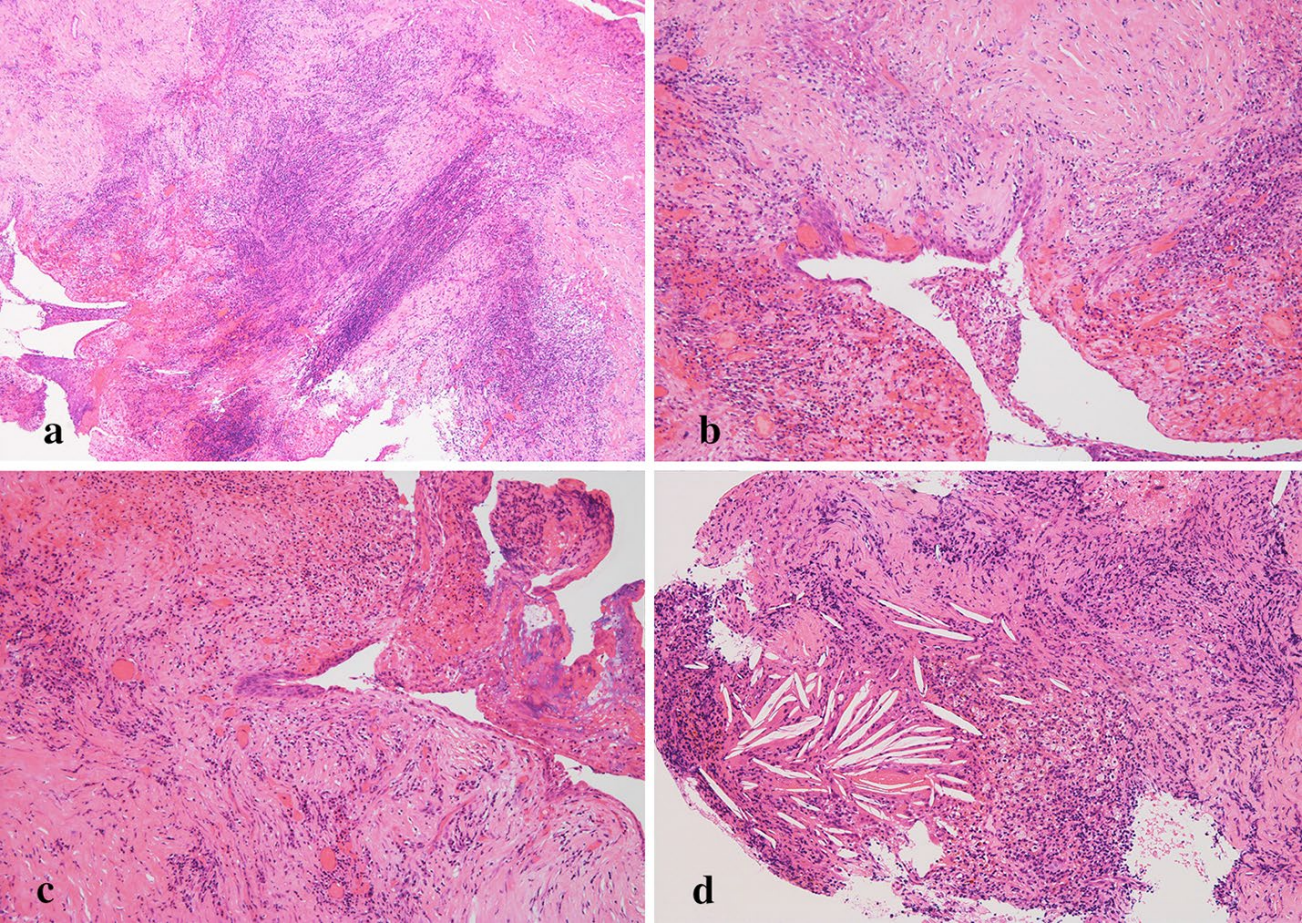
Case 2. **a** Photomicrograph showing massive inflammatory granulation, original magnification ×50. **b**, **c** Smooth transition from single squamous epithelium to stratified squamous epithelia, original magnification ×100. **d** Evident cholesterin clefts are seen, indicating repeated hemorrhage in the cyst, original magnification ×100. Hematoxylin and eosin stain

Surgery was performed through the extended transsphenoidal approach. Intraoperative finding disclosed that the tough and extremely fibrous lesion had adhered directly to the right optic nerve and encased the ipsilateral A1-2 junction of the anterior cerebral artery. The lesion was removed en-bloc together with the infiltrated right rectal gyrus. Subtotal removal was achieved. Postoperative histological examination disclosed smooth transition from single squamous epithelium to stratified squamous epithelia. The diagnosis was established as Rathke’s cleft cyst with significant squamous metaplasia. However, marked sub-epithelial infiltration of inflammatory cells was seen, and massive inflammatory granulation had formed. Most infiltrated cells were lymphocytes and plasma cells, frequently seen in neuroglial tissues (Fig. 6).

Rapid deterioration caused by immune-response abnormality was suspected and pulse-dose steroid therapy was begun with daily methylprednisolone 1000 mg for 3 days and gradual reduction to daily dexamethasone 0.5 mg with a short bridge of hydrocortisone. She was discharged after 43 postoperative days with disappearance of cerebral edema and thin linear enhanced lesion in the suprasellar cistern. She remained in good condition with supplement of daily triamcinolone 2 mg after 31 months.

## Discussion

A question would be raised about mechanism of this aggressive clinical course. Some investigators may presume possible influence of ethanol fixation. We basically performed aspiration of cyst content and subsequent ethanol fixation for Rathke’s cleft cysts unless cyst wall was widely communicated with CSF spaces, by temporary attachment of cotton flakes soaked with pure ethanol [10, 11]. Both cases in this report did not communicate with CSF spaces in the first operation and collapse of the cyst was achieved once, although rapid re-deterioration was occurred after long intervals of 6 months in case 1, and 18 months in case 2.

Severe Rathke’s cleft cyst may be indicated by squamous metaplasia histology [3, 5, 10–13]. Both our patients had significant squamous metaplasia and marked sub-epithelial inflammation. Most infiltrated cells were lymphocytes and plasma cells, which suggests the involvement of chronic inflammatory processes in this phenomenon. Long-term subclinical inflammation may be part of the mechanism in these cases of severe Rathke’s cleft cyst [14].

Although hydrocortisone or prednisolone is more popular for the treatment of hypopituitarism dexamethasone has unique character in itself. Biological half-life is long and binding with glucocorticoid receptor is strong, so down regulation of receptor is believe to be less inducible than other steroid agents [15]. For this reason some clinicians prefer to this agent especially for long-term usage.

## Conclusion

Two cases of Rathke’s cleft cysts with intraparenchymal infiltration and extensive cerebral edema were treated. Long-term subclinical inflammation may be the mechanism of this aggressive clinical course, so postoperative steroid administration should be reduced prudently, and careful follow-up imaging is essential in cases of Rathke’s cleft cysts with abnormal histological findings.

## Abbreviations

CSF: cerebrospinal fluid
MR: magnetic resonance
